# All-Optical Electrophysiology in hiPSC-Derived Neurons With Synthetic Voltage Sensors

**DOI:** 10.3389/fncel.2021.671549

**Published:** 2021-05-28

**Authors:** Francesca Puppo, Sanaz Sadegh, Cleber A. Trujillo, Martin Thunemann, Evan P. Campbell, Matthieu Vandenberghe, Xiwei Shan, Ibrahim A. Akkouh, Evan W. Miller, Brenda L. Bloodgood, Gabriel A. Silva, Anders M. Dale, Gaute T. Einevoll, Srdjan Djurovic, Ole A. Andreassen, Alysson R. Muotri, Anna Devor

**Affiliations:** ^1^Department of Pediatrics/Rady Children’s Hospital San Diego, School of Medicine, University of California, San Diego, La Jolla, CA, United States; ^2^Department of Neurosciences, University of California, San Diego, La Jolla, CA, United States; ^3^Department of Cellular and Molecular Medicine, School of Medicine, University of California, San Diego, La Jolla, CA, United States; ^4^Department of Biomedical Engineering, Boston University, Boston, MA, United States; ^5^Division of Biological Sciences, Section of Neurobiology, University of California, San Diego, La Jolla, CA, United States; ^6^Division of Mental Health and Addiction, Oslo University Hospital, NORMENT, KG Jebsen Centre for Neurodevelopmental Disorders, Institute of Clinical Medicine, University of Oslo, Oslo, Norway; ^7^Department of Chemistry and Molecular and Cell Biology, University of California, Berkeley, Berkeley, CA, United States; ^8^Department of Bioengineering, University of California, San Diego, La Jolla, CA, United States; ^9^Department of Radiology, University of California, San Diego, La Jolla, CA, United States; ^10^Faculty of Science and Technology, Norwegian University of Life Sciences, Ås, Norway; ^11^Department of Physics, University of Oslo, Oslo, Norway; ^12^Department of Medical Genetics, Oslo University Hospital, Oslo, Norway; ^13^NORMENT, Department of Clinical Science, University of Bergen, Bergen, Norway; ^14^Kavli Institute for Brain and Mind and Halıcıoglu Data Science Institute, University of California, San Diego, La Jolla, CA, United States; ^15^Center for Academic Research and Training in Anthropogeny (CARTA), La Jolla, CA, United States; ^16^Department of Radiology, Harvard Medical School, Athinoula A. Martinos Center for Biomedical Imaging, Charlestown, MA, United States

**Keywords:** stem cells, voltage imaging, BeRST-1, phenotyping, optogenetics

## Abstract

Voltage imaging and “all-optical electrophysiology” in human induced pluripotent stem cell (hiPSC)-derived neurons have opened unprecedented opportunities for high-throughput phenotyping of activity in neurons possessing unique genetic backgrounds of individual patients. While prior all-optical electrophysiology studies relied on genetically encoded voltage indicators, here, we demonstrate an alternative protocol using a synthetic voltage sensor and genetically encoded optogenetic actuator that generate robust and reproducible results. We demonstrate the functionality of this method by measuring spontaneous and evoked activity in three independent hiPSC-derived neuronal cell lines with distinct genetic backgrounds.

## Introduction

Traditionally, neuronal electrical properties have been evaluated using intracellular electrophysiological recordings, such as whole-cell patch clamp (Reyes, 2019). Recent advances in optical microscopy and optogenetics offer a new and complementary experimental paradigm of “all-optical electrophysiology” (Hochbaum et al., 2014; Kiskinis et al., 2018), where genetically encoded voltage indicators (Yang and St-Pierre, 2016) and optogenetic (OG) actuators (Deisseroth, 2015) are combined for all-optical stimulation and readout of neuronal activity (Emiliani et al., 2015). While whole-cell patch recordings remain unsurpassed in their ability to quantify small currents and membrane potential changes in single neurons, all-optical electrophysiology is less labor intensive and better suited for simultaneous measurements from multiple neurons. Applied to biological model systems derived from human induced pluripotent stem cells (hiPSCs) (Takahashi et al., 2007; Yu et al., 2007), these methods open unprecedented opportunities for non-invasive phenotyping of neurons and neuronal networks with unique genetic backgrounds of individual patients (Marchetto et al., 2010; Brennand et al., 2011; Mertens et al., 2015). For example, in hiPSC-derived neurons, high sensitivity of whole-cell patch recordings was leveraged to assess synaptic activity and ion channel properties across developmental stages (Brennand et al., 2011; Belinsky et al., 2014; Fink et al., 2017), while all-optical electrophysiology was used for high-throughput characterization of action potential waveforms and spiking patterns (Kiskinis et al., 2018).

In prior all-optical electrophysiology studies, the voltage sensor and OG actuator were co-expressed using viral transduction (Hochbaum et al., 2014; Kiskinis et al., 2018; Bando et al., 2019). Here, we sought to establish an alternative protocol that uses synthetic voltage sensors (Peterka et al., 2011; Miller, 2016; Walker et al., 2020a,b) that can be easily delivered to cell cultures. We demonstrated the functionality of this method by measuring spontaneous and OG-induced activity in three independent hiPSC-derived neuronal cell lines with distinct genetic backgrounds.

### Results

All-optical electrophysiology requires that the voltage sensor and OG actuator are spectrally orthogonal in order to minimize the crosstalk, i.e., avoiding excitation of the voltage sensor by light that controls the OG actuator and vice versa (Hochbaum et al., 2014; Emiliani et al., 2015; Kiskinis et al., 2018). Specifically, blue-shifted OG actuators such as CheRiff can be combined with red-shifted voltage sensors (Zhou et al., 2007; Huang et al., 2015). We chose Berkeley Red Sensor of Transmembrane potential (BeRST-1) as one of the available red shifted synthetic voltage indicators that can be safely delivered to all cells and offers fast response kinetics and high sensitivity to single spikes (Huang et al., 2015). We combined it with photoactivation of the OG actuator CheRiff that has been previously used in all-optical electrophysiology experiments (Hochbaum et al., 2014). CheRiff is a genetically encoded actuator that requires viral transduction (see section “Methods”); however, the impact of transduction with lentiviruses carrying a CheRiff-EGFP expression cassette alone on cell viability was not significant in our hands. We also co-loaded cells with a synthetic calcium (Ca^2+^) indicator Oregon Green BAPTA-1 AM (OGB1) to perform Ca^2+^ imaging prior to voltage imaging to identify active fields of view (FOVs).

#### Optimization of Imaging Protocol

**FIGURE 1:**
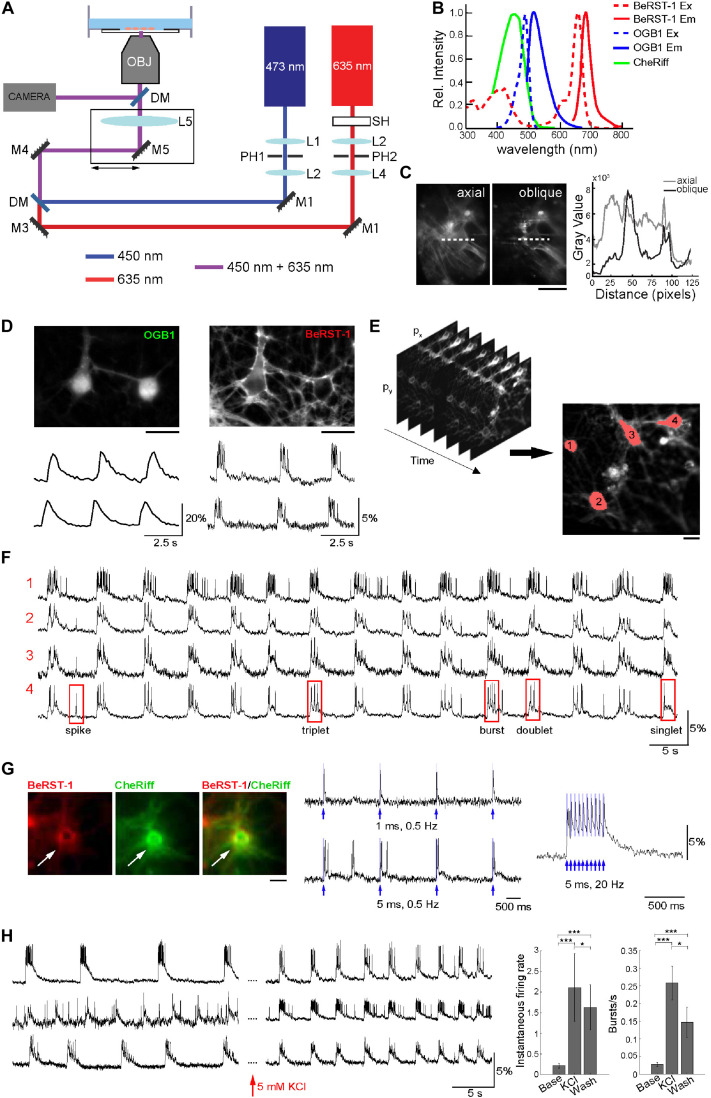
Optimization of the imaging protocol in primary neurons. **(A)** Imaging Setup. The 635-nm and 473-nm laser beams were collimated and spatially filtered through a combination of lenses (L1–L4) and pinholes (PH1 and PH2). A shutter SH was used to block the 635-nm beam when not acquiring data; the 473-nm beam was modulated using an analog signal. The beams were combined by a dichroic mirror DM1 and focused onto the rear focal plane of the objective via a lens LS. The lens and mirror M5 were translated together in the plane orthogonal to the optical axis to offset the illumination enter the objective producing oblique illumination. Fluorescence was directed to a camera for detection of BeRST-1 or OGB1 signals through a dichroic mirror an emission filter. **(B)** Overlaid excitation (dashed line) and emission (solid line) spectra of BeRST-1 (red) (Huang et al., 2015) and OGB1 (blue, reproduced from Fisher Scientific) and action spectrum of CheRiff (green) (Hochbaum et al., 2014). **(C)** Comparison of BeRST-1 fluorescence profile with axial and oblique illumination (gray and black lines, respectively). Scale bar, 10 μm. **(D)** Primary neurons co-labeled with OGB1 (left) and BeRST-1 (right). Scale bar, 10 μm. Time-courses of spontaneous Ca^2+^ and voltage activity extracted from these two neurons are shown below the respective OGB1 and BeRST-1 images. **(E)** Segmentation of single-neuron ROIs. Scale bar, 10 μm. **(F)** Extraction of single-neuron voltage time-courses. The four traces in (F) correspond to four different neurons segmented in panel **(E)**. **(G)** All-optical electrophysiology with BeRST-1 in CheRiff-expressing neurons. Left: Primary neurons stained with BeRST-1 (red) and expressing CheRiff-EGFP (green). Right: Voltage response to OG stimulation of varying frequency and duration. **(H)** High K^+^ increases spiking activity in primary neurons. Left: Voltage time-courses of three representative neurons in normal imaging buffer and after perfusion with 5 mM KCl. Right: Instantaneous firing rate and bursting rate at baseline (Base, *n* = 9 neurons), after 5 min of perfusion with 5 mM KCl (KCl, *n* = 9 neurons) and after washing off high K^+^ (Wash; *n* = 25). Error bars indicate mean ± SD; unpaired Student’s *t*-test (*p < 0.05; **p < 0.01; ***p < 0.001).

The imaging setup consisted of a body of an inverted epifluorescence microscope with a custom illumination light path (Figure 1A). The illumination was provided by a pair of diode lasers at 635 and 473-nm for simultaneous BeRST-1 imaging and CheRiff actuation or, alternatively, sequential imaging of BeRST-1 and OGB1 (Figure 1B) (see section “Methods”). The laser beams were collimated and combined by a dichroic mirror. To reduce background fluorescence due to out-of-focus excitation we implemented a custom solution for oblique angle illumination (Werley et al., 2017) significantly improving the signal-to-background ratio (SBR) (see section “Methods”, Figure 1C and Supplementary Figure 1).

To troubleshoot our imaging protocol, we used primary cultures of rat dissociated hippocampal neurons at 14–23 days *in vitro* (DIV) that had robust and synchronous spiking activity (see section “Methods” and Supplementary Figures 2A–F). Co-labeling with OGB1 was critical for quick and efficient evaluation of the level of spiking activity and for choosing FOVs for subsequent voltage imaging. Further, cytosolic OGB1 staining facilitated visual inspection of the cell culture including cell morphology, density, and connectivity (Figure 1D). We imaged spontaneous voltage activity continuously for ∼3 min using FOVs of ∼ 300 × 150 μm acquired at ∼500 Hz, (2.2 ms exposure, 25 mW/cm^2^ laser power). Consecutive acquisition periods were separated by at least 2 min where the 635-nm illumination was blocked by a mechanical shutter. Under this regime, we observed virtually no photobleaching (see Supplementary Figure 2G). The size of FOV was limited by the camera rate. With a 20X objective, our FOVs included up to 12 neurons. Image analysis and segmentation were adapted from published methods (Mukamel et al., 2009; Hochbaum et al., 2014). The algorithm was used to extract several key parameters describing firing properties of individual neurons including the shape of the action potential (AP), number of spikes, plateau depolarizations, and different types of bursting behavior (Figures 1E,F and section “Methods”). Primary neurons at 17 DIV had AP duration of 10.1 ± 7.14 ms (mean ± SD; *n* = 27 neurons, 635 APs) at 30°C in the imaging chamber (see section “Methods” and Supplementary Figure 3).

Next, we transducted primary neurons with lentivirus carrying CheRiff-EGFP expression cassette under the CamKIIα promoter for all-optical electrophysiology (Figure 1G). BeRST-1 has a second, smaller excitation peak around 420 nm. In our hands, OG stimulation at 450 nm (that is commonly used for excitation of blue OG actuators) resulted in excitation of BeRST-1 seen as an increase in the voltage signal following the shape of the 450-nm laser pulse (not shown). Switching to a 473-nm laser eliminated this cross talk. For troubleshooting the protocol, we used 5 mM potassium chloride or 50 μM picrotoxin (gamma aminobutyric acid antagonist) as a means for inducing high-level spiking activity (Figure 1H and Supplementary Figure 4). This procedure was used to evaluate the ability of neurons to produce spikes in viable neurons “on demand” during the troubleshooting of the protocol irrespective of the level of CheRiff expression.

#### Optimization of hiPSC-Derived Cell Culture Protocol

**FIGURE 2:**
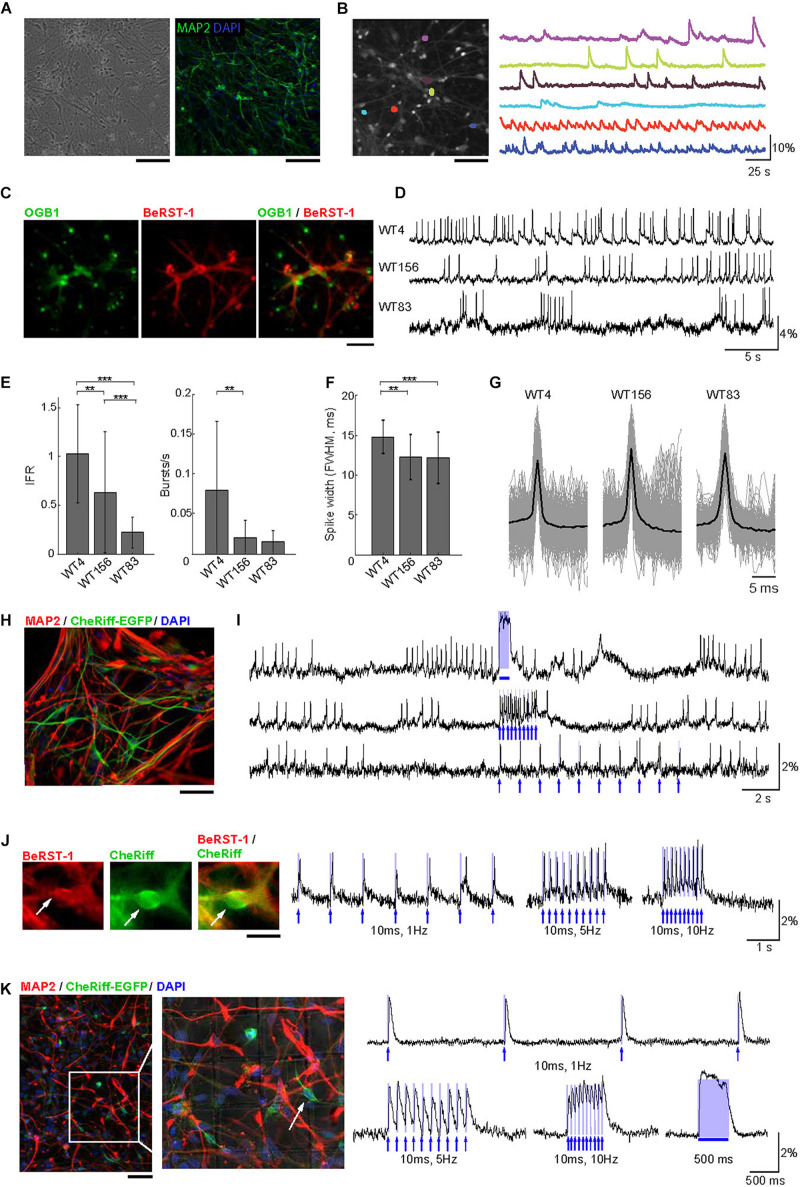
Voltage imaging and all-optical electrophysiology in human neurons. **(A)** Monolayer cultures of human neurons. Left: Transmitted light image of cell culture after 8 weeks of differentiation. Right: cell culture after 10 weeks of differentiation immunostained for MAP2 and DAPI. Scale bars, 200 μm. **(B)** Ca^2+^ imaging in human neurons. Left: A representative FOV with OGB1-loaded neurons. Right: Color-coded Ca^2+^ time-courses from six ROIs corresponding to individual neurons. Scale bar, 200 μm. **(C)** Human neurons stained with OGB1 (left, green), BeRST-1 (middle, red), and overlay of the two signals (right). **(D)** Voltage imaging in human neurons with BeRST-1. Representative time-courses of spontaneous voltage activity in three hiPSC-derived control cell lines showing different levels of activity and distinct firing patterns. **(E)** Quantification of instantaneous firing rate and bursting rate across the three cell lines (*n* = 24 neurons in line WT4; *n* = 36 in line WT156; *n* = 41 in line WT83). Data are shown as mean ± SD; unpaired Student’s *t*-test (*p < 0.05; **p < 0.01; ***p < 0.001). **(F)** AP waveforms for the three control cell lines (line WT4: *n* = 16 neurons, 383 APs; line WT156: *n* = 12, 2349 APs; line WT83: *n* = 8, 783 APs. **(G)** Distribution of the AP duration [full width at half maximal (FWHM) amplitude] for the APs shown in panel **(E)**: line WT4, 14.8 ± 2.1 ms; line WT156, 12.3 ± 2.8 ms; line WT83, 12.2 ± 3.2 ms. Data are shown as mean ± SD. **(H)** Immunostaining of human neurons for MAP2 (red) and EGFP (green). The nuclei were counterstained with DAPI (blue). Scale bar, 350 μm. **(I)** OG stimulation in spontaneously active CheRiff-expressing control neurons (line WT4). The top trace shows activity evoked by stimulation with a continuous 500-ms OG stimulus; the two bottom traces show stimulation with 5-ms long light pulses of different frequencies (1 and 10 Hz). The timing of OG stimulation is indicated in blue. **(J)** Evoked depolarization and spiking in human neurons with low spontaneous activity. Left: A representative human neuron expressing CheRiff-EGFP (green) and stained with BeRST-1 (red). Scale bar, 15 μm. Right: spiking induced by 10-ms long OG stimulation of increasing frequency (1, 5, and 10 Hz). **(K)** Left: *Post hoc* immunolabeling with MAP2 (red), and EGFP (green); the nuclei are counterstained with DAPI (blue). The localization grid is visible in the zoomed-in image. Scale bar, 500 μm. Right: all-optical electrophysiology from one neuron prior to fixation. The timing of OG stimulation is indicated in blue.

Next, we translated our imaging protocol to hiPSC-derived neuronal cell cultures. The success rate of our experiments critically depended on obtaining healthy, active, and spatially uniform monolayer cultures of differentiated neurons (Figure 2A). To this end, we modified a previously published procedure (Calabrese et al., 2019) aiming to promote the growth of healthy, well-adhered, sparse monolayer cultures with limited clustering and minimal presence of dead cells (see section “Methods” and Supplementary Figure 5). Our protocol started with the initial 4–5 weeks of neural progenitor cells (NPC) differentiation followed by replating and further 3–4 weeks of differentiation before transduction with the lentivirus carrying CheRiff-EGFP (see section “Methods” and Supplementary Figure 5). Compared to the protocol of Calabrese et al. (2019), we delayed the replating procedure to prevent proliferation of undifferentiated cells and improved the dissociation technique (see section “Methods”). All-optical electrophysiology was performed 7–10 days after the viral transduction when robust EGFP florescence indicated successful expression of the transgene (Supplementary Figure 5).

#### Voltage Imaging in hiPSC Derived Neurons With BeRST-1

Similar to primary neurons in Figure 1, we used OGB1 for quick evaluation of the level of activity in human neurons (Figure 2B) taking advantage of larger FOVs achievable with Ca^2+^ imaging due to a tradeoff between FOV and imaging speed (Kulkarni and Miller, 2017). Co-labeling of hiPSC-derived cultures with BeRST-1 and OGB1 (Figure 2C) was also used for visual inspection for remaining undifferentiated NPCs or overgrowth of glia providing feedback for optimization of the cell culture protocol (see section “Methods” and Supplementary Figure 5). We applied our protocol to three independent hiPSC-derived neuronal cell lines with distinct genetic backgrounds (WT4, WT83, and WT156). In agreement with previous reports (Shi et al., 2012; Paavilainen et al., 2018), spontaneous activity was low and heterogeneous during early development increasing after ∼8 weeks of differentiation [withdrawal of basic fibroblast growth factor (bFGF) from the medium, see section “Methods”]. Interestingly, we observed clear differences in the firing patterns between the three cell lines (Figures 2D,E). Most noticeably, the firing rate varied between 1.03 ± 0.50-Hz in line WT4 (*n* = 24 neurons) and 0.23 ± 0.16-Hz in line WT83 (*n* = 41 neurons) (Figure 2E). The AP waveform did not differ significantly across the cell lines (Figures 2F,G).

Next, we transducted human neurons with lentivirus carrying CheRiff-EGFP expression cassette under the CamKIIα promoter for all-optical electrophysiology (Figure 2H). We observed higher heterogeneity in EGFP fluorescence in human neurons compared to primary neurons, likely reflecting variable levels of CheRiff-EGFP expression in human neurons at different stages of maturation. Photoactivation of CheRiff robustly induced depolarization and spiking irrespective of the level of spontaneous activity (Figures 2I,J).

The heterogeneity of firing properties within a cell line, as indicated by the error bars in Figure 2E, could be at least in part due to differences in neuronal cell types. In contrast to genetically encoded voltage sensors, synthetic probes such as BeRST-1 cannot be targeted to specific cell types. This limitation, however, can be mitigated by fixation and *post hoc* immunolabeling, as long as the fixation and labeling procedure does not distort the cellular network allowing for registration with live images. *Post hoc* labeling and image registration have been performed on cell cultures in the past (Hanson et al., 2010; Walker et al., 2020b) but not in the context of voltage imaging in human neurons, although Hochbaum et al. (2014) have used immunolabeling for sodium channels in their study that combined CheRiff with a genetically encoded voltage sensor. As a proof of principle, we plated neurons in imaging dishes with an imprinted grid. Individual neurons were targeted for OG stimulation and voltage imaging following by *in situ* fixation and immunolabeling immediately after the imaging session (see section “Methods”). With this procedure, we were able to find FOVs and specific neurons used for live imaging (Figure 2K). Although the majority (∼90%) of neurons produced with our culturing protocol were glutamatergic (Zhang et al., 2016; Marchetto et al., 2017), in the future, this protocol may help addressing cell-type-specific neuronal activity while circumventing the need for genetic encoding of voltage sensors.

### Conclusion

In summary, voltage imaging with synthetic probes such as BeRST-1 combined with genetically encoded OG actuators provides a robust paradigm for all-optical electrophysiology in hiPSC-derived neurons and neuronal networks possessing unique genetic background of individual patients. In the present study, this protocol has proven to be very effective offering high quality imaging readouts. While synthetic probes such as BeRST-1 indiscriminately label all membranes, the cell type of imaged neurons can be identified *post hoc* using immunostaining and image registration. The present observation of varying firing patterns across the considered control cell lines supports the use of isogenic controls whenever possible, as previously suggested (Iakoucheva et al., 2019). Indeed, isogenic controls are commonly used in hiPSC-based studies of point mutations (Chailangkarn et al., 2012; Kiskinis et al., 2018). However, engineering such controls may pose a challenge in case of polygenic brain disorders. We envision that the same protocol, including fixation after all-optical-electrophysiology, will be used in future studies for single-cell-resolved nuclei-isolated RNAseq or *in situ* RNA hybridization from imaged neurons providing a link from the functional phenotype to the underlying gene expression profile.

## Materials and Methods

### Imaging Setup

We engineered our imaging setup around a body of an inverted Olympus IX71 epifluorescence microscope. Illumination was provided by a pair of CW diode lasers at 635-nm (500 mW, Opto Engine LLC) and 473-nm (100 mW, Cobolt 06-MLD). The laser beams were collimated, spatially filtered, and combined by a dichroic mirror (Semrock, FF01-370/36-25). After the mirror, co-aligned beams were directed toward a lens (AC508-400-A-ML, *f* = 400 mm) focusing the light onto the rear focal plane of a high numerical aperture objective [Olympus UPlanFL N 20X, numerical aperture (NA) = 0.5 (air) or Olympus UPlanFL N 40X/NA = 1.30 (oil)]. The lens (and a mirror in front of the lens) was translated in the plane orthogonal to the optical axis displacing the focal spot off the axis in the rear focal plane of the objective. This produced oblique illumination, where the laser light propagated at a low angle close to the bottom of the culture dish after refracting at the glass-water interface (Werley et al., 2017). By restricting the fluorescence to a region of the specimen near the bottom of the dish, oblique illumination reduced the background improving the signal-to-background ratio (SBR) (Fiolka, 2016). The angle of incidence of the light onto the specimen depended of the amount of offset of the lens allowing fine adjustment for achieving the best results. Oblique illumination was in particular important in cases with overlapping cells and out-of-focus debris.

The 635-nm beam was used for BeRST-1 imaging with intensity at the sample of 25 W/cm^2^. A shutter was placed in front of the 635-nm beam to control the illumination time of the sample without the need for turning the laser off, which would lead to power instability. The 473-nm beam was used for CheRiff actuation with intensity at the sample of 5–10 mW/cm^2^. Laser power of the 473-nm beam was controlled by an analog square signal provided by a computer-controlled National Instruments Data Acquisition (DAQ) board. The same 473-nm beam was used for OGB1 imaging with intensity at the sample of 8 mW/cm^2^ in experiments that did not involve OG stimulation.

Berkeley Red Sensor of Transmembrane potential fluorescence was filtered at 736 ± 64 nm and collected using a scientific sCMOS camera (Zyla 4.2 Plus, Andor) operated at 300–500 Hz, 2.2 ms exposure, 1X gain, FOV of 200 × 150 μm. OGB1 fluorescence was filtered at 535 ± 25 nm and collected with the same camera operated at 50 Hz. The emission filters were swapped in between voltage and Ca^2+^ imaging. Data were collected in epochs of ∼3 min for imaging of spontaneous neuronal activity and 30 s for all-optical electrophysiology. Consecutive data acquisition epochs were separated by at least 2 min where the 635-nm illumination was shuttered off.

### Primary Neuronal Cultures

All animal procedures were performed in accordance with the University of California San Diego Institutional Animal Care and Use Committee and complied with all relevant ethical regulations for animal research. Dissociated hippocampal neuronal cultures were derived from wild-type Sprague Dawley rat pups of both sexes at postnatal days P0–P1. The cell cultures were prepared as previously described (Goslin et al., 1991), and plated at a density of 130 cells per mm^2^ on laminin-coated 35-mm MatTek dishes (MatTek CatP35G-0-14-C). Neurons were grown in Neurobasal-A media (ThermoFisher Scientific Cat10888022) supplemented with Glutamax (ThermoFisher Scientific Cat35050061), Pen/Strep (ThermoFisher Scientific Cat10378016), and B27 supplement (ThermoFisher Scientific Cat17504044).

### Human Induced Pluripotent Stem Cell-Derived Neuronal Cultures

All iPSC were provided by the Muotri lab. Cortical NPCs were differentiated from hiPSC as described previously (Griesi-Oliveira et al., 2015). To differentiate NPCs to cortical neurons, NPCs were dissociated with Accutase (StemCell Technologies), plated on a 10-cm tissue culture dishes coated with 10 μg/ml poly-L-ornithine (PLO; Sigma, P3655) and 2.5 μg/ml mouse laminin (1 mg, Invitrogen 23017-015), and then cultured for 2 weeks in NPC medium (DMEM/F12 supplemented with 1% penicillin-streptomycin, N2 NeuroPlex (Gemini Bio-products), NeuroCult SM1 (Stem-Cell technologies), and 20 ng/mL basic fibroblast growth factor (bFGF; Life Technologies). When NPCs reached 90% confluency, NPCs were differentiated into cortical neurons by bFGF withdrawal. Differentiating cells were fed with NPC medium every 3 days. hiPSC-derived cultures were differentiated for 4–5 weeks to allow growth of healthy neurons, extension of long processes, and formation of densely interconnected networks. Neurons were then dissociated and replated on 35-mm imaging plates with glass bottoms, as described below, and kept differentiating for a further 3–4 weeks with excellent cell survival, cell recovery and connectivity re-growth.

### Cell Dissociation and Replating

To dissociate and replate mature neurons that had already established long neurites and a complex connectivity network, cells were dissociated with Accutase (StemCell Technologies). After 40 min of incubation, the dense network of cells that had lifted from the plate was mechanically dissociated by slowly pipetting up and down using a 5-ml wide-tipped pipette. The culture was then incubated for an additional 10 min. Next, DMEM/F12 medium was added to the plate to block the enzymatic reaction and further gentle mechanical trituration was carried out, first with a 1,000-μl pipette and then with a 200-μl pipette to gradually loose and dissociate the thick networks of neurites and cell clusters. The dissociated culture was strained through a 70-μm cell strainer to remove clumps, and then centrifuged for 4 min at 900 rpm. The resulting cell pellet was re-suspended in Media2 [Neurobasal media (Life Technologies) supplemented with GlutaMAX (Life Technologies), 1% Gem21 NeuroPlex, 1% MEM non-essential amino acids (NEAA, Life technologies), and 1% Penicillin streptomycin] supplemented with 1% fetal bovine serum (FBS, Gemini Bio-products), 1 μg/ml of laminin and 0.1% ROCK (Rho kinase) inhibitor (Y-27632, Fisher Scientific). Cells were replated on a 14-mm cover slip of a 35-mm imaging plates coated with 100 μg/ml PLO and 5 μg/ml laminin to reach a final density of 0.3 million cells, with a typical cell viability of 80–90%. After 6 h, additional M2 medium was added to allow dead cells to lift and be aspirated away. The neurons were then fed with fresh M2 medium supplemented with 5 μM cytosine arabinoside (AraC) (Sigma-Aldrich) to prevent proliferation of glial cells underneath the neurons. After 2 days of AraC exposure, the cells were fed with fresh M2 medium; feeding was repeated once per week with M2 medium (half media exchange).

### Gene Delivery

CheRiff was delivered via lentiviral transduction after transferring to the imaging dish. Lentivirus was produced by VectorBuilder (Cyagen) from CamKIIα-CheRiff plasmid (Hochbaum et al., 2014). DRH313: FCK-CheRiff-eGFP was a gift from Adam Cohen (Addgene plasmid #51693^1^; RRID:Addgene_51693). Primary neurons were transduced at 6 DIV and used for experiments at 14–23 DIV. hiPSC-derived neurons were transduced 7–10 days prior to imaging. On the transfection day, 4–12 μL of the CheRiff lentivirus were combined with 200 μL of conditioned medium from the culture to allow the cells benefit from their secreted growth factors (Yang et al., 2017). Following an overnight incubation, an additional 2 ml of M2 medium were added. After one more day of incubation, the plates were washed and replenished with fresh M2 medium. hiPSC-derived neurons were fed with antibiotic-free M2 medium for 1 week prior to transduction, which resulted in higher expression levels and lower cell death.

### Loading of Optical Probes

Berkeley Red Sensor of Transmembrane potential was produced as previously described (Huang et al., 2015) and stored as 5 mM in DMSO. OGB1-AM (O-6807, Invitrogen, 50 μg) was first dissolved in 4 μl of 20% pluronic in DMSO (F-127, Invitrogen); 80 μl of imaging buffer (MgCl_2_ 1 mM, NaCl 130 mM, KCl 3 mM, CaCl_2_ 1 mM, Glucose 10 mM, and HEPES 10 mM; pH 7.4) were added to yield the final concentration of 0.5 mM OGB1-AM. Both primary and hiPSC-derived cultures were incubated in the imaging buffer with the final concentration of 5 μM BeRST-1 and 5 μM OGB1-AM for ∼20 min immediately prior to the imaging session. We did not use OGB1 in experiments involving OG stimulation. During this time, cells were kept within an incubator at 37°C. Next, cells were washed and replenished with fresh imaging buffer. Data were acquired at 30°C. Temperature was maintained by continuous perfusion with a heated imaging buffer (see below). Careful temperature maintenance was important, because it affected the level of activity, firing pattern, and the AP waveform (Supplementary Figure 3).

### Perfusion System

Cultures were perfused with imaging buffer for the entire duration of the experiment. We used a diamond-shaped bath (Warner Instruments LLC) incorporated into the imaging dish to provide a laminar flow across the culture. The speed of perfusion was controlled with a peristaltic pump (Peri-Star, WPI) at 2 ml/min. An in-line heater (SC-20, Warner Instruments) was positioned in close proximity of the imaging chamber to maintain the temperature of the imaging buffer within the dish at 30°C. The heater was calibrated with a thermo-probe inserted into the dish. The imaging buffer containing high K^+^ (5 mM) or picrotoxin (50 μM) were washed in using computer-controlled microfluidic valves (VC-6, Warner Instruments).

### Image Processing and Signal Analysis

The protocol for image analysis was adapted from Mukamel et al. (2009) and Hochbaum et al. (2014). In brief, images were filtered in time and space to improve the signal-to-noise ratio (SNR). Then, a combination of principal component analysis (PCA) and independent component analysis (ICA) was used to identify regions of interest (ROIs) corresponding to individual neurons and extract corresponding single-neuron voltage time-courses. The time-courses were computed as percent change relative to the baseline (Δ*F*/*F*_0_). Single-neuron Δ*F*/*F*_0_ time-courses were computed as described in Hochbaum et al. (2014).

For spike detection, we adapted an algorithm based on a non-linear energy operator (NEO) that was previously used for extracellular electrophysiological recording to emphasize spike-like signals (Mukhopadhyay and Ray, 1998). The NEO signal was calculated as *s*(*t*)^2^ – *s*(*t* – 1) × *s*(*t* + 1), where *s*(*t*) is single-neuron voltage time-course Δ*F*/*F*_0_. Peaks were identified in the NEO signal using a MATLAB peak-seek routine. All peaks below a three standard deviations of the NEO signal were discarded. To avoid double counting when the same AP crossed the threshold twice due to high frequency noise in the recording, we set a hard limit of <20 ms on the time between two spikes (inter-spike-interval, ISI).

To obtain AP waveforms, we extracted segments of voltage traces in a window of 300 ms centered on a detected AP peak. The waveforms were then aligned on the peak, and the waveform parameters were extracted as previously described (Bean, 2007; Trainito et al., 2019). The spike duration was quantified by calculating the full width at half-maximal amplitude (FWHM).

We used a custom MATLAB routine to distinguish individual APs from those riding on top of plateau depolarizations including one (singlet), two (doublet), and three (triplet) APs. A burst was defined as any sequence of four or more APs with mean inter-spike-interval (ISI) shorter than 250 ms (Chen et al., 2009). The algorithm computed instantaneous firing frequency, bursting rate, ISI, IBI, and burst duration.

### *Post hoc* Immunolabeling

Neurons were replated on 50-μm grids patterned on the bottom of 35-mm MatTek dishes (MatTek CatP35G-0-14-C) previously coated with 100 μg/ml PLO and 5 μg/ml laminin. Immediately after live imaging, cells were fixed *in situ* with 4% paraformaldehyde (PFA, Core Bio Services) for 30 min at room temperature. The cells were then washed three times in phosphate buffered saline (PBS), permeabilized with 0.2% Triton X-100 in PBS for 15 min and blocked with 2% bovine serum albumin (BSA, Gemini Bio-products) in PBS for 2 h at room temperature. Primary antibodies (chicken anti-MAP2, Abcam ab5392, 1:500; rabbit anti-GFP, Molecular Probes A-21311, 1:1,000) were diluted in blocking buffer (2% BSA in PBS). Cells were incubated with the primary antibodies overnight at 4°C. The following day cells were washed three times in PBS, and incubated with the secondary antibodies coupled to Alexa Fluor 488, 555, and 647 (Life Technologies, 1:1,000 in blocking buffer) for 1 h at room temperature. Cells were then washed three times in PBS to remove the secondary antibodies, and DAPI (1:10,000 in PBS) was added for 30 min at room temperature to counterstain cell nuclei. Cells were then washed again three times in PBS. Finally, the PBS was aspirated, and the cells were submerged in a drop of ProLong Gold anti-fade mountant (Life Technologies) and covered with a glass coverslip for subsequent analysis.

### Statistics

All results are expressed as the mean ± standard deviation from mean. For evaluation of statistical significance, datasets were compared using unpaired 2-tailed Student’s *t* test. *p* values < 0.05 were considered significant.

Our goal was to establish a protocol for obtaining healthy/active monolayer and spatially uniform cultures that were critical for the overall success of our experiments. In common practice, cells are typically cultured in high-density to boost the mutual benefit of secreted neurotrophic factors (Yang et al., 2017). These high density cultures tend to form large clusters of overlapping neurons. Single-photon fluorescent imaging, however, does not possess depth sectioning. Therefore, this application works best in monolayers of cells, where a possibility for signal cross-talk between adjacent/overlapping neurons is insignificant. Although this cross-talk can be mitigated in part by using oblique illumination (Werley et al., 2017) and computational unmixing (Mukamel et al., 2009; Hochbaum et al., 2014), these remedies are not very effective in over-confluent cultures, in particular when clustered cells fire synchronously. In our hands, high density cultures also exhibited a high rate of cell death due to viral transduction leading to poor expression of the OG actuator CheRiff.

Achieving monolayer cultures with hiPSCs was noticeably more difficult compared to primary cultures due to a well-recognized lengthy process of transition from neural progenitors cells (NPC) to neuronal networks requiring careful control of growth and maturation (Calabrese et al., 2019). Specifically, neurons growing for a long period of time in the same dish commonly form clustered, interconnected networks producing robust patterns of network level activity (Segev et al., 2001; Wagenaar et al., 2006). While this may be desirable for multi-electrode array (MEA) studies (Biffi et al., 2013), such networks are suboptimal for single-photon fluorescence imaging due to the abovementioned problem of signal cross-talk. In addition, after more than 5 weeks of growth in the same dish, we observed that hiPSC-derived cell cultures had the following three properties aversive for optical imaging:

(i) They easily detached from the substrate when handled.
(ii) They often contained a layer of dead cells attached to healthy neurons. Dead cells sequestered optical probes generating strong background fluorescence.
(iii) They contained NPCs that remained undifferentiated, continued to divide and, in many cases, dominated over differentiated neurons competing with them for the growth substrate. Under these conditions, NPCs could also deprive differentiated neurons of their nutrients.

To mitigate these problems, we modified a published cell culture protocol (Calabrese et al., 2019) resulting in well-adhered, relatively sparse and uniform cultures with limited cell clustering. Our protocol included the following critical steps:

(i) The initial 4–5 weeks of NPC differentiation in 10-cm Petri dishes.
(ii) Replating the cells into 35-mm imaging plates only after mature neurons with robust and long interconnections were formed.
(iii) Further 2–3 weeks of differentiation to allow recovery, re-growth of connections and neuronal maturation.
(iv) Transduction with lentivirus carrying CheRiff-EGFP expression cassette.
(v) All-optical electrophysiology performed 7–10 days after transfection when robust EGFP fluorescence indicated successful expression of the transgene.

Depending on the cell line, the maturation time-course varied. Therefore, the timing of replating and transfection was determined based on observation of the culture growth and connectivity. Best results were achieved when replating occurred prior to slowing down of growth and connectivity and a decrease in cell viability.

#### IPS Culture Protocol

##### Culturing NPCs in 10-cm dishes

1. 2 days before thawing or splitting NPCs, coat 10-cm petri dishes with PLO and mouse laminin.
  – Dissolve PLO in sterile water to make a stock solution (10 mg/mL). Store this stock at −20°C. Dilute PLO 1:1,000 in water to yield a concentration of 10 μg/mL. Apply the coating directly to the target dishes using 5 mL of PLO solution. Incubate at 37°C and 5% CO_2_ overnight.
  – Retrieve coated dishes, aspirate the PLO solution and rinse twice with sterile water.
  – Thaw laminin at 4°C and quickly add to Dulbecco’s PBS (dPBS) to avoid aggregation of laminin and uneven coating. Prepare a solution of 2.5 μg/mL mouse laminin in dPBS. Coat the entire dish surface with 5 mL laminin solution. Incubate at 37°C and 5% CO_2_ overnight.

2. Retrieve coated dishes from the incubator, aspirate the laminin solution, rinse once with dPBS, and plate NPCs.
3. Culture NPCs for about 1 week (timing depends on the specific cell line) in NPC medium [DMEM/F12 supplemented with 1% penicillin-streptomycin, N2 NeuroPlex, NeuroCult SM1 and 20 ng/mL basic fibroblast growth factor (bFGF)]. Allow the culture to reach about 90% confluency.
4. Feed NPCs culture with fresh NPC medium every other day.

##### Differentiating neurons in 10-cm dishes

1. On the first day of neural differentiation, retrieve NPC plates from the incubator, fully aspirate the NPC medium from the edge of the dish, rinse the culture with 5 ml dPBS; then add fresh neuronal medium (NPC medium without bFGF) supplemented with 0.1% ROCK inhibitor.
2. After 36 h, fully aspirate the medium and add fresh neuronal medium supplemented with 0.1% ROCK inhibitor.
3. After 36 h, fully aspirate and add fresh neuronal medium.
4. Change neuronal medium every other day for the first 10 days of differentiation making sure to aspirate and add medium gently from the edge of the dish.
5. After 10 days of differentiation, change half the neuronal medium every 3–4 days to improve neural growth in conditioned media (released neurotrophic factors) and to prevent mechanical stress and cell detachment.
6. Allow neurons to differentiate for 4–5 weeks until they reach neuronal maturation and form a densely interconnected network of long processes.

##### Replating differentiated neurons onto imaging dishes

1. 2 days before replating, coat the 14-mm glass coverslips of 35-mm imaging dishes with PLO and mouse laminin.
  – Dissolve PLO in sterile water to make a stock solution (10 mg/mL). Store this stock at −20°C. Dilute PLO 1:100 in distilled water to yield a concentration of 50 μg/mL. Apply the coating directly to the target glass area of the imaging dishes using 400 μl of PLO solution. Incubate at 37°C and 5% CO_2_ overnight.
  – Retrieve coated dishes, aspirate the PLO solution and rinse four times with sterile water.
  – Prepare a solution of 5 μg/mL mouse laminin in dPBS. Coat the glass coverslips with 400 μl laminin solution. Incubate at 37°C and 5% CO_2_ overnight.

2. On the replating day, retrieve the 10-cm NPC plates from the incubator, aspirate the neuronal medium, rinse once with dPBS, add 4 mL of Accutase, and incubate at 37°C and 5% CO_2_ for 40 min. Check the cells every 10–15 min to evaluate the dissociation process.
3. After 40 min of dissociation, retrieve the dishes from the incubator. A dense mesh of cells and processes must have lifted from the plate. Gently dissociate mechanically by slowly pipetting up and down using a 5-ml wide-tipped pipette.
4. Incubate the culture for an additional 10 min.
5. Retrieve the dishes from the incubator, add 8 ml of neuronal medium to block the enzymatic reaction and gently apply further mechanical trituration with a 1,000-μl pipette to gradually loose and dissociate the thick network of neurites and cell clusters.
6. Strain the dissociated culture through a 70-μm cell strainer to remove clumps.
7. Centrifuge for 4 min at 900 rpm.
8. Re-suspend the resulting cell pellet in Media2 (Neurobasal media supplemented with GlutaMAX, 1% Gem21 NeuroPlex, 1% MEM non-essential amino acids, and 1% Penicillin streptomycin) supplemented with 1% fetal bovine serum (FBS), 1 μg/ml of laminin and 0.1% ROCK inhibitor.
9. Count cells and assess cell viability in hemocytometer.
10. Retrieve previously coated 35-mm plates from the incubator. Aspirate the laminin solution, rinse with dPBS, and replate cells onto the target 14-mm glass coverslip to reach a final density of 0.3 million cells, at a typical cell viability of 80–90%. Incubate at 37°C and 5% CO_2_ for 6 h.
11. After 6 h, retrieve the imaging dishes from the incubator, add M2 medium to allow dead cells to lift and be aspirated away.
12. Feed the replated neurons with fresh M2 medium supplemented with 5 μM cytosine arabinoside (AraC) to prevent proliferation of glial cells underneath the neurons.
13. After 2 days of AraC exposure, feed the cells with fresh M2 medium.
14. Change half the M2 medium once per week.

## Data Availability Statement

The original contributions presented in the study are included in the article/Supplementary Material, further inquiries can be directed to the corresponding authors.

## Ethics Statement

The animal study was reviewed and approved by University of California San Diego Institutional Animal Care and Use Committee.

## Author Contributions

FP data curation, software, formal analysis, validation, investigation, visualization, methodology, writing–original draft, and writing–review and editing. SS resources, investigation, visualization, methodology, and writing–review and editing. CT resources, data curation, investigation, methodology, and writing–review and editing. MT resources, software, investigation, methodology, and writing–review and editing. MV methodology and writing–review and editing. EC, XS, and EM resources and writing–review and editing. IA software. BB resources. GS software and writing–review and editing. ADa, GE, SD, and OA funding acquisition and writing–review and editing. ARM conceptualization, resources, supervision, funding acquisition, methodology, project administration, and writing–review and editing. ADe conceptualization, data curation, supervision, funding acquisition, writing–original draft, project administration, and writing–review and editing. All authors contributed to the article and approved the submitted version.

## Conflict of Interest

ARM is a co-founder and has equity interest in TISMOO, a company dedicated to genetic analysis and brain organoid modeling focusing on therapeutic applications customized for autism spectrum disorder and other neurological disorders with genetic origins. The terms of this arrangement have been reviewed and approved by the University of California San Diego in accordance with its conflict of interest policies. The remaining authors declare that the research was conducted in the absence of any commercial or financial relationships that could be construed as a potential conflict of interest.

## Footnotes

1 http://n2t.net/addgene:51693

## Supplementary Material

Supplementary Figure 1 Oblique-angle epi-fluorescence. **(A)** Principle of oblique-angle epi-fluorescence. Oblique-angle epifluorescence microscopy uses illumination beams directed at subcritical angles to light the target cell components near the glass barrier. Only the fluorophores (such as NIR BeRST fluorescent molecules) in a thin subsurface layer of the target specimen are excited, resulting in more selective imaging and less background signal. In our setup, the mirror M5 directs the co-aligned red and blue beams toward the lens L5 focusing the light onto the rear focal plane of a high numerical aperture objective. L5 is translated in the plane orthogonal to the optical axis to displace the focal spot off the axis in the rear focal plane of the objective. **(B)** Photographs showing the implemented oblique-angle illumination scheme in our optical setup. After the mirror (M5), co-aligned beams were directed toward lens L5 (AC508-400-A-ML, *f* = 400 mm) focusing the light onto the rear focal plane of a high numerical aperture objective [Olympus UPlanFL N 20X, numerical aperture (NA) = 0.5 (air) or Olympus UPlanFL N 40X/NA = 1.30 (oil)].

Supplementary Figure 2 Voltage imaging across developmental stages in primary neurons. **(A)** In primary neurons, the level of activity and degree of network synchronization increased from DIV 7 to DIV 23. This was accompanied by the emergence of network bursts. Top: Representative voltage time-courses recorded at 7, 14, 17, and 23 DIV. Bottom: raster plots showing spike timing for a subset of the recorded neurons. **(B–E)** Quantification of spiking behavior at 7, 14, 17, and 23 DIV. A burst is a sequence of 4 or more spikes with inter-spike-interval lower than 250 μs. Data are shown as mean ± SD; unpaired Student’s *t*-test (7 DIV, *n* = 8 neurons; 14 DIV, *n* = 26 neurons; 17 DIV, *n* = 32 neurons; 23 DIV, *n* = 34 neurons). IBI, inter-burst interval. **(F)** The AP duration decreases with neuronal maturation. The AP duration was quantified as the full width at half maximal amplitude (FWHM) at 7 DIV (*n* = 5 neurons, 316 APs), 14 DIV (*n* = 4, 635 APs), and 17 DIV (*n* = 5, 1279 APs). Data are shown as mean ± SD; unpaired Student’s *t*-test. These observations are in agreement with previous work in hippocampal primary cultures (Penn et al., 2016). **(G)** Under our imaging conditions, we observed virtually no photobleaching of BeRST-1 for the 3 min of continuous illumination with the 635-nm laser at 25 mW/cm^2^ laser power. Top: a representative raw voltage trace. Bottom: the output of the data processing algorithm.

Supplementary Figure 3 Temperature effects. **(A)** Temperature alters intrinsic membrane excitability, response properties and firing rates (Thompson et al., 1985). Therefore, careful maintenance of temperature within the imaging chamber was critical for robustness and reproducibility of results. Representative voltage time-courses obtained from a primary neuron at room temperature (22°C, left) or under perfusion with heated imaging buffer (30°C, right) show a dramatic increase in the firing rate as well as generation of bursts and plateau depolarization. **(B)** These effects were also present in hiPCS-derived cultures. Representative voltage time-courses from a human neuron at room temperature (22°C, left) or under perfusion with heated imaging buffer (32°C, right) show a sharp increase in the firing frequency. **(C)** In both primary and human neurons, the AP duration was temperature-dependent. For human neurons, the AP duration, quantified as the full width at half maximum amplitude, decreased from 45.8 ± 26.9 to 10.6 ± 4.5 ms with an increase of the bath temperature from 22°C (*n* = 11 neurons, 1028 APs) to 32°C (*n* = 6 neurons, 7,000 APs). **(D)** Under the same conditions, the instantaneous firing frequency increased from 0.18 ± 0.18 to 7.12 ± 5.85 Hz, and the bursting frequency–from 0.02 ± 0.02 to 0.07 ± 0.05 Hz. Data are shown as mean ± SD; unpaired Student’s *t*-test.

Supplementary Figure 4 Effect of picrotoxin on spontaneous firing of primary neurons. Perfusion with 50 μM picrotoxin increases the activity in rat hippocampal neurons. Voltage time-courses obtained from six representative neurons show spontaneous activity prior to pharmacological stimulation (left) and an increase in firing and bursting activity under 50 μM picrotoxin (PTX, right).

Supplementary Figure 5 Generation of monolayer cultures of hiPSC-derived neurons. **(A)** Transmitted light image of NPCs (scale bar, 200 μm) plated on 10-cm Petri dishes. **(B)** Transmitted light image of NPCs culture that reached a confluency of 90%, which is the point when bFGF was withdrawn to start differentiation. Scale bar, 200 μm. **(C)** Transmitted light image of developing human neurons after 4 weeks of differentiation with long and robust connections. Scale bar, 200 μm. **(D)** hiPSC-derived neuronal cultures after ∼8 weeks of differentiation in 10-cm Petri dishes, without replating. Transmitted light images show formation of clusters (i), detached cells (ii, left) and clumps of cells on top of each other (ii, right). Clumps of cells from human neurons, which have been differentiating for 8 weeks, stained with BeRST-1 and loaded with OGB1 (iii). Scale bars, 50 μm. **(E)** Replating into 35-mm imaging plates at week 4–5 of differentiation allows formation of homogenous and sparse neuronal cultures. The culture was immune-stained with MAP2 (red), the nuclei were counterstained with DAPI (blue). Scale bar, 50 μm. **(F)** Monolayer cultures facilitate expression of CheRiff-EGFP via lentiviral transduction. The epifluorescence images show representative examples of CheRiff-EGFP-expressing neurons 10 days after the viral transfection. **(G)** Human neurons after 10 weeks of differentiation, which were replated at week 4. The transmitted light image shows a homogenous, monolayer culture cells with no clustering. The immunostaining image indicates mature neurons expressing MAP2 (red) and CheRiff-EGFP (green). The nuclei were counterstained with DAPI (blue). Scale bar, 200 μm. **(H)** hiPSC neurons were imaged between week 9 and 10 of differentiation. Top: Epifluorescence images of cells loaded with OGB1 and stained with BeRST-1. Bottom: three representative BeRST-1 traces of spontaneous activity.
