# Supplementary figures and images for: All-Optical Electrophysiology in hiPSC-Derived Neurons With Synthetic Voltage Sensors
Source: Front Cell Neurosci. 2021 May 28;15:671549. doi: 10.3389/fncel.2021.671549 (PMC8193062; doi:10.3389/fncel.2021.671549)

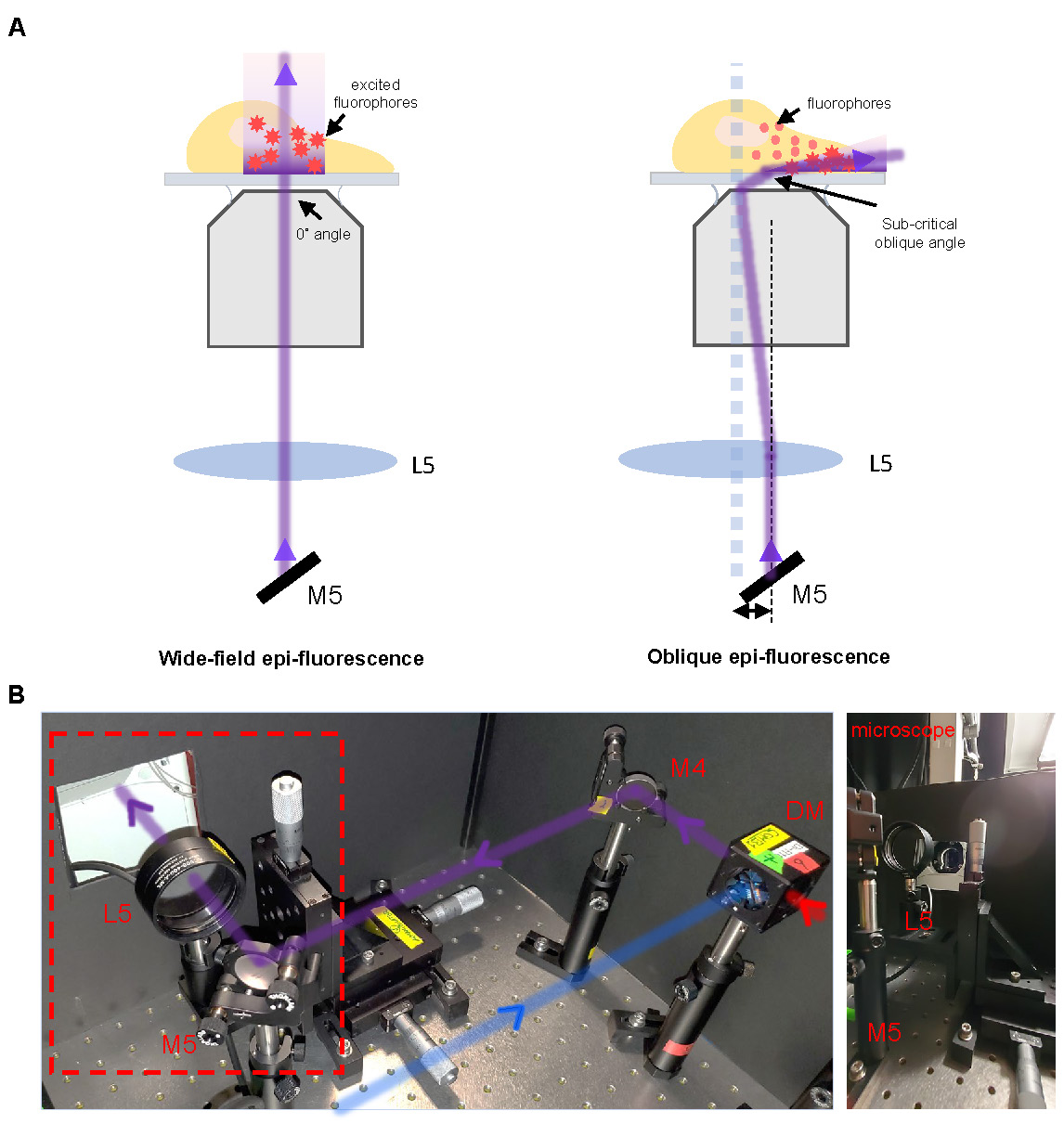

Supplement: Supplementary Figure 1 — Oblique-angle epi-fluorescence. (A) Principle of oblique-angle epi-fluorescence. Oblique-angle epifluorescence microscopy uses illumination beams directed at subcritical angles to light the target cell components near the glass barrier. Only the fluorophores (such as NIR BeRST fluorescent molecules) in a thin subsurface layer of the target specimen are excited, resulting in more selective imaging and less background signal. In our setup, the mirror M5 directs the co-aligned red and blue beams toward the lens L5 focusing the light onto the rear focal plane of a high numerical aperture objective. L5 is translated in the plane orthogonal to the optical axis to displace the focal spot off the axis in the rear focal plane of the objective. (B) Photographs showing the implemented oblique-angle illumination scheme in our optical setup. After the mirror (M5), co-aligned beams were directed toward lens L5 (AC508-400-A-ML, f = 400 mm) focusing the light onto the rear focal plane of a high numerical aperture objective [Olympus UPlanFL N 20X, numerical aperture (NA) = 0.5 (air) or Olympus UPlanFL N 40X/NA = 1.30 (oil)]. [file Image_1.jpg]

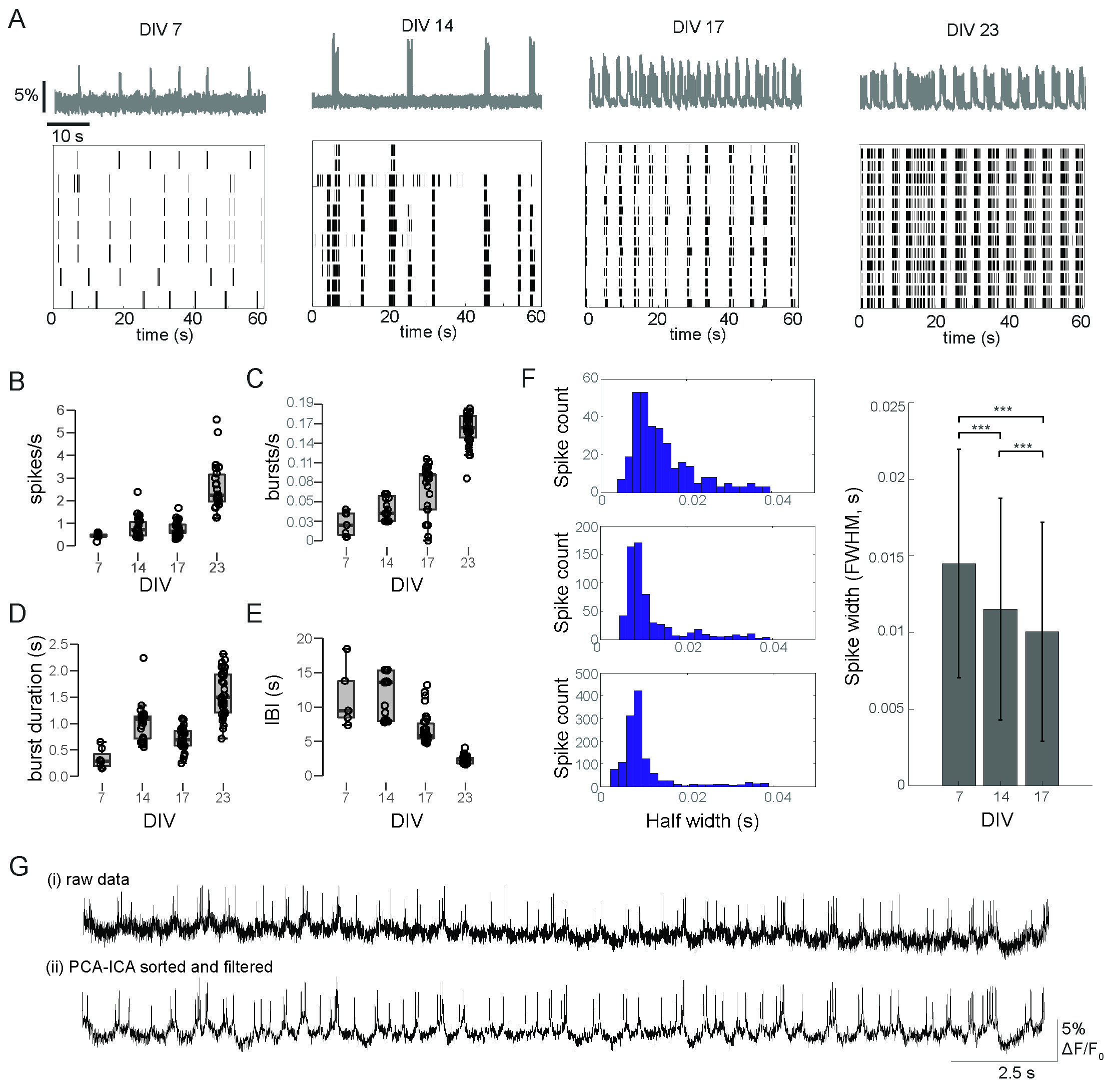

Supplement: Supplementary Figure 2 — Voltage imaging across developmental stages in primary neurons. (A) In primary neurons, the level of activity and degree of network synchronization increased from DIV 7 to DIV 23. This was accompanied by the emergence of network bursts. Top: Representative voltage time-courses recorded at 7, 14, 17, and 23 DIV. Bottom: raster plots showing spike timing for a subset of the recorded neurons. (B–E) Quantification of spiking behavior at 7, 14, 17, and 23 DIV. A burst is a sequence of 4 or more spikes with inter-spike-interval lower than 250 μs. Data are shown as mean ± SD; unpaired Student’s t-test (7 DIV, n = 8 neurons; 14 DIV, n = 26 neurons; 17 DIV, n = 32 neurons; 23 DIV, n = 34 neurons). IBI, inter-burst interval. (F) The AP duration decreases with neuronal maturation. The AP duration was quantified as the full width at half maximal amplitude (FWHM) at 7 DIV (n = 5 neurons, 316 APs), 14 DIV (n = 4, 635 APs), and 17 DIV (n = 5, 1279 APs). Data are shown as mean ± SD; unpaired Student’s t-test. These observations are in agreement with previous work in hippocampal primary cultures (Penn et al., 2016). (G) Under our imaging conditions, we observed virtually no photobleaching of BeRST-1 for the 3 min of continuous illumination with the 635-nm laser at 25 mW/cm2 laser power. Top: a representative raw voltage trace. Bottom: the output of the data processing algorithm. [file Image_2.jpg]

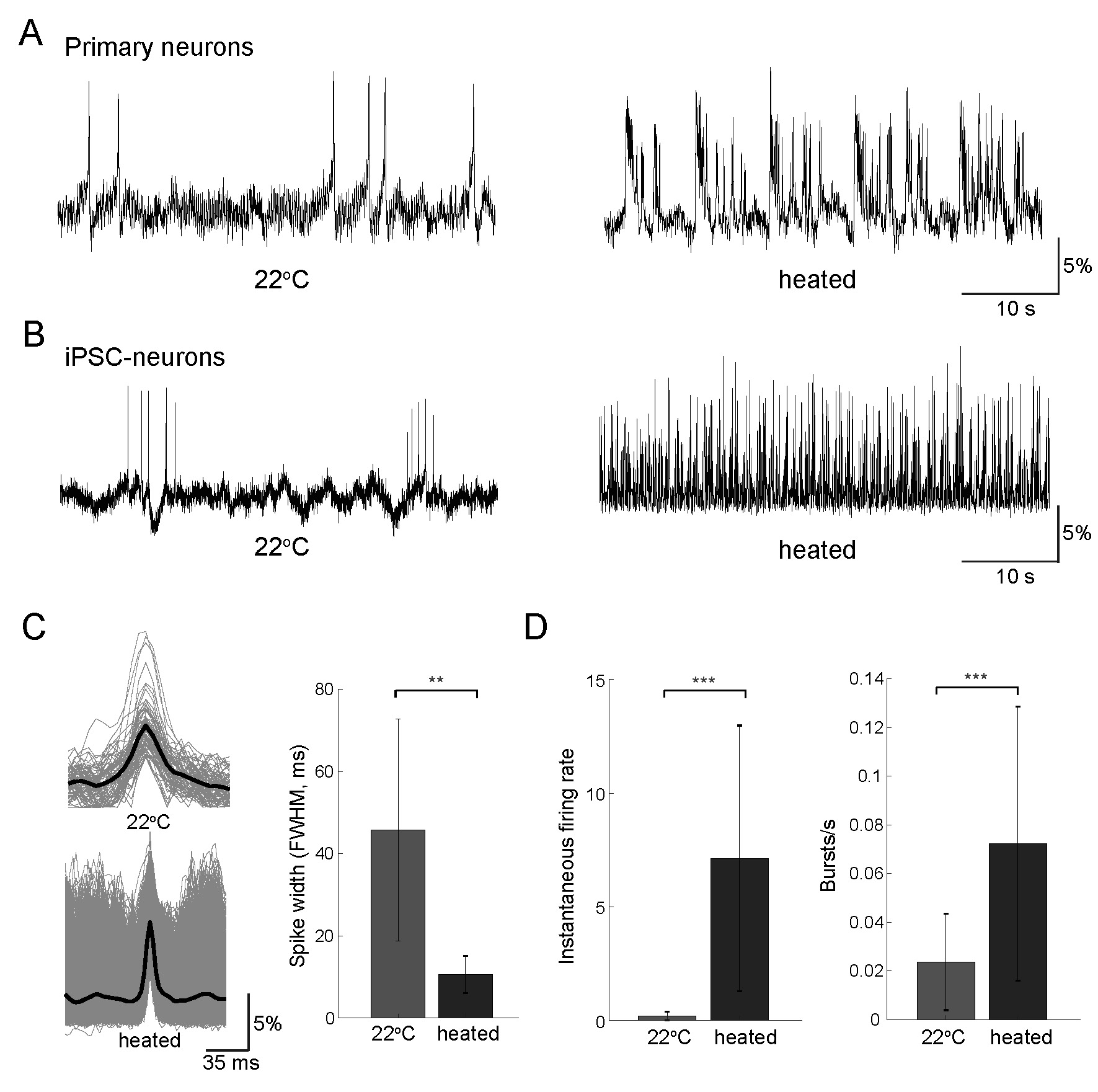

Supplement: Supplementary Figure 3 — Temperature effects. (A) Temperature alters intrinsic membrane excitability, response properties and firing rates (Thompson et al., 1985). Therefore, careful maintenance of temperature within the imaging chamber was critical for robustness and reproducibility of results. Representative voltage time-courses obtained from a primary neuron at room temperature (22°C, left) or under perfusion with heated imaging buffer (30°C, right) show a dramatic increase in the firing rate as well as generation of bursts and plateau depolarization. (B) These effects were also present in hiPCS-derived cultures. Representative voltage time-courses from a human neuron at room temperature (22°C, left) or under perfusion with heated imaging buffer (32°C, right) show a sharp increase in the firing frequency. (C) In both primary and human neurons, the AP duration was temperature-dependent. For human neurons, the AP duration, quantified as the full width at half maximum amplitude, decreased from 45.8 ± 26.9 to 10.6 ± 4.5 ms with an increase of the bath temperature from 22°C (n = 11 neurons, 1028 APs) to 32°C (n = 6 neurons, 7,000 APs). (D) Under the same conditions, the instantaneous firing frequency increased from 0.18 ± 0.18 to 7.12 ± 5.85 Hz, and the bursting frequency–from 0.02 ± 0.02 to 0.07 ± 0.05 Hz. Data are shown as mean ± SD; unpaired Student’s t-test. [file Image_3.jpg]

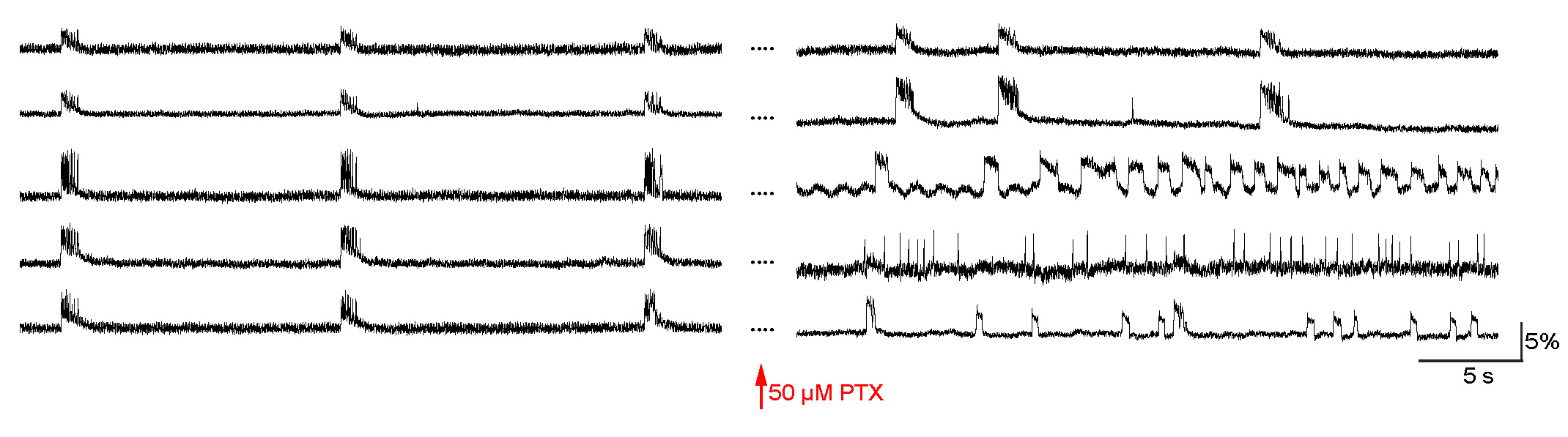

Supplement: Supplementary Figure 4 — Effect of picrotoxin on spontaneous firing of primary neurons. Perfusion with 50 μM picrotoxin increases the activity in rat hippocampal neurons. Voltage time-courses obtained from six representative neurons show spontaneous activity prior to pharmacological stimulation (left) and an increase in firing and bursting activity under 50 μM picrotoxin (PTX, right). [file Image_4.jpg]

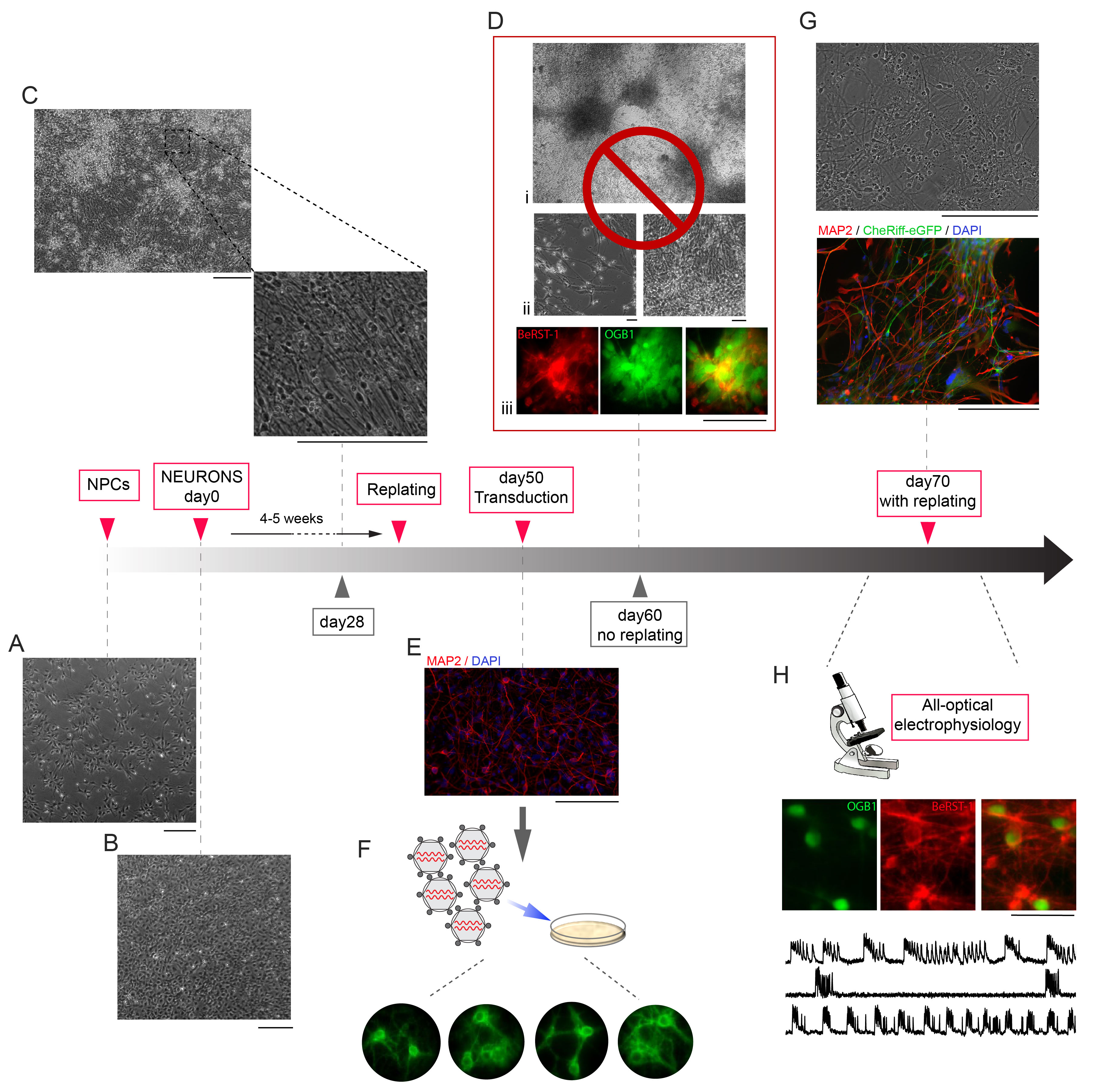

Supplement: Supplementary Figure 5 — Generation of monolayer cultures of hiPSC-derived neurons. (A) Transmitted light image of NPCs (scale bar, 200 μm) plated on 10-cm Petri dishes. (B) Transmitted light image of NPCs culture that reached a confluency of 90%, which is the point when bFGF was withdrawn to start differentiation. Scale bar, 200 μm. (C) Transmitted light image of developing human neurons after 4 weeks of differentiation with long and robust connections. Scale bar, 200 μm. (D) hiPSC-derived neuronal cultures after ∼8 weeks of differentiation in 10-cm Petri dishes, without replating. Transmitted light images show formation of clusters (i), detached cells (ii, left) and clumps of cells on top of each other (ii, right). Clumps of cells from human neurons, which have been differentiating for 8 weeks, stained with BeRST-1 and loaded with OGB1 (iii). Scale bars, 50 μm. (E) Replating into 35-mm imaging plates at week 4–5 of differentiation allows formation of homogenous and sparse neuronal cultures. The culture was immune-stained with MAP2 (red), the nuclei were counterstained with DAPI (blue). Scale bar, 50 μm. (F) Monolayer cultures facilitate expression of CheRiff-EGFP via lentiviral transduction. The epifluorescence images show representative examples of CheRiff-EGFP-expressing neurons 10 days after the viral transfection. (G) Human neurons after 10 weeks of differentiation, which were replated at week 4. The transmitted light image shows a homogenous, monolayer culture cells with no clustering. The immunostaining image indicates mature neurons expressing MAP2 (red) and CheRiff-EGFP (green). The nuclei were counterstained with DAPI (blue). Scale bar, 200 μm. (H) hiPSC neurons were imaged between week 9 and 10 of differentiation. Top: Epifluorescence images of cells loaded with OGB1 and stained with BeRST-1. Bottom: three representative BeRST-1 traces of spontaneous activity. [file Image_5.jpg]
